# Effects of fluorine modification on the photocatalytic hydrogen production performance of TiO_2_

**DOI:** 10.3389/fchem.2025.1621188

**Published:** 2025-06-05

**Authors:** Jie Hu, Xianhao Shan, Shan Wu, Pengfei Sun, Zhengyuan Gao, Zhong Ren, Xiangchao Feng, Shuai Wang

**Affiliations:** ^1^ School of Materials and Science, Chongqing Jiaotong University, Chongqing, China; ^2^ School of Mechatronics and Vehicle Engineering, Chongqing Jiaotong University, Chongqing, China; ^3^ Institute of Aerospace Intelligence and Innovation, Academy of Aerospace System and Innovation, Beijing, China

**Keywords:** surface-adsorbed fluorination, lattice-doped fluorination, TiO_2_, Ti-F bonds, photocatalytic hydrogen production

## Abstract

As an efficient and environmentally friendly photocatalyst, TiO_2_ has garnered significant interest among researchers. However, the rapid recombination of photogenerated carriers leads to the inhibition of its photocatalytic activity. Fluorine modification has been proven to be an effective method to improve the photocatalytic performance of TiO_2_, leading to a multitude of research reports on this subject. Surface fluorine adsorption or lattice fluorine doping can deftly modulate the surface chemical attributes and electronic configuration of the TiO_2_ photocatalyst, thereby amplifying its functional performance. The role of fluorine atoms coordinated with different number titanium atoms (terminal Ti_1_-F, bridging Ti_2_-F and Ti_3_-F) are also discussed. This paper provides a minireview of various aspects of fluorine-modified TiO_2_, including its classification (surface-adsorbed fluorination, lattice-doped fluorination and Ti_x_-F) and characterization techniques (X-ray photoelectron spectroscopy and solid-state nuclear magnetic resonance). Finally, this treatise elucidates the mechanistic impact of fluorine modification on the photocatalytic hydrogen production performance of TiO_2_.

## 1 Introduction

Titanium dioxide (TiO_2_) is one of the most extensively utilized photocatalysts due to its excellent stability, cost-effectiveness and eco-friendliness (Nishiyama et al., 2021; Bhom and Isa, 2024; Wen et al., 2024). The low efficiency of photogenerated carrier separation and transport limits the wide application of TiO_2_ (Cheng et al., 2024; Zhao et al., 2025). To enhance the photocatalytic activity of TiO_2_, numerous modification studies have been undertaken, including noble metal deposition (Li et al., 2021), doping (Zhang et al., 2019) and heterojunction construction (Ma et al., 2019). A pivotal discovery made by Lai et al., in 1993 revealed that adjusting the ratio of hydrofluoric acid (HF) to fluoride ion in fluorinated reaction solutions could alter the band edge potential of TiO_2_ (Lai et al., 1993). This finding established fluorine modification as an effective strategy to improve the photocatalytic performance of TiO_2_ due to enhanced surface acidity, stronger adsorption of reactant molecules, additional Ti^3+^ self-doping, and stabilized {001} facets (Yang et al., 2008). After substituting O atoms or surface hydroxyl groups, fluorine introduced into TiO_2_ is usually classified as surface-adsorbed fluorine or lattice-doped fluorine (Wu and Schmuki, 2023). Moreover, according to the different number of titanium atoms coordinated with fluorine, Wang et al. and Hu et al. proposed that the F atoms doped in fluorinated TiO_2_ system by a variety of chemical bonds: terminal Ti_1_-F bond (F1s), bridging fluorine F2c (Ti_2_-F) and 3-coordinated fluorine F3c (Ti_3_-F), where the x in Ti_x_-F represents the number of titanium atoms bonded to this fluorine atom (Wang et al., 2013). However, due to the complex types of Ti-F bonds in fluorinated TiO_2_, the mechanism of carrier separation and transport is unclear, the precise control of Ti-F bonds is difficult, the mechanism of fluorination reaction is not clear, and the understanding of the structure-property relationship is insufficient. There is an urgent need for follow-up and cooperation in related theoretical research. This minireview aims to summarize the impact of fluorine modification on the photocatalytic hydrogen production performance of TiO_2_ through a comprehensive review of relevant literature. The discussion will encompass aspects such as classification of TiO_2_ fluorination, characterization of fluorine species, and effect of fluorine on the photocatalytic hydrogen generation performance of TiO_2_.

## 2 Classification of TiO_2_ fluorination

The fluorination route influences the physicochemical properties and photocatalytic performance of F-TiO_2_. Generally, the modification of TiO_2_ with fluorine encompasses both surface-adsorbed fluorination and lattice-doped fluorination (Zulfiqar et al., 2021). Surface-adsorbed fluorine is typically achieved through post-treatment fluorination via a ligand exchange between F^−^ ions and the surface functional groups of TiO_2_. The realization of fluorine doping in the internal phase lattice of materials often requires the introduction of fluorine in the preparation process of TiO_2_ for *in-situ* synthesis. Herein, we will briefly describe the fluorination principle and fluorine species of these fluorination method, as well as the detailed structures and descriptions of corresponding examples.

### 2.1 Surface-adsorbed fluorination

**FIGURE 1:**
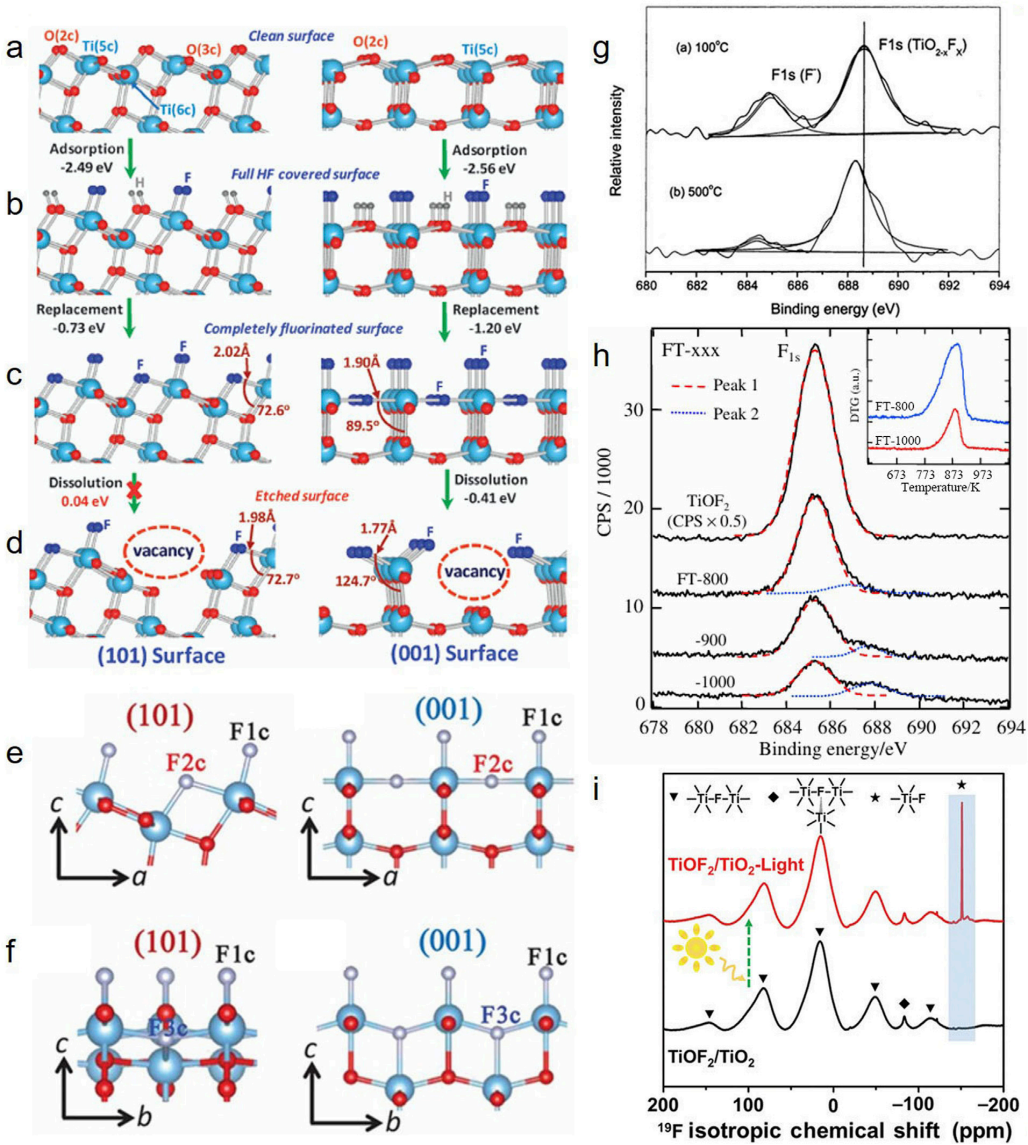
DFT (density functional theory) calculated reaction energies and structures for different stages of HF interaction with single crystal anatase TiO_2_(101) (left) and (001) (right) surfaces: **(a)** Clean surfaces; **(b)** full HF-covered surfaces; **(c)** complete fluorinated surfaces; **(d)** etched surfaces; **(e)** fluorinated surface with lattice F2c atoms; **(f)** fluorinated surface with lattice F3c atoms. All structures are optimized structures (Wang et al., 2011; Wang et al., 2013). XPS spectra of **(g)** F_1s_ spectra of F-doped TiO_2_ (Yu et al., 2002) and **(h)** F_1s_ spectra of FT powder and pure TiOF_2_ (Li et al., 2005); **(i)** ^19^F NMR spectra of TiOF_2_/TiO_2_ (Hu et al., 2020a).

Surface fluorinated TiO_2_ material can be easily obtained by simple ligand exchange between F- and the surface hydroxyl group (OH^−^) through exposing TiO_2_ photocatalyst to a mild aqueous solution containing F^−^ (NaF, NH_4_F, ILs-F) (Park and Choi, 2004; Wang et al., 2008; Lin et al., 2020). After being immersed in NaF aqueous solution, the coordination unsaturated surface Ti^4+^ ions in TiO_2_ will combine with water to form various hydrates, and the chemisorbed water molecules will dissociate to ≡Ti-OH to produce surface hydroxyl. Ligand exchange occurs between F^−^ and ≡Ti-OH to complete the adsorption of fluorine on the TiO_2_ surface to form Ti_1_-F (Park and Choi, 2004). Compared with simple exchange ≡Ti-OH, etching TiO_2_ surface with HF can change the surface properties more strongly (Wang et al., 2008). When low concentration HF is etched, F^−^ not only replaces the end hydroxy-group on the surface but also the lattice oxygen. However, F^−^ does not penetrate into the interior of the TiO_2_ lattice, and the substitution of lattice oxygen only occurs on the surface (Wang et al., 2008). Some studies also believe that during HF etching, HF dissociates and adsorbates on the clean TiO_2_ surface during surface fluoridation. When the adsorption site on the surface is completely occupied by fluorine, the exposed hydroxyl group on the surface will be replaced by fluorine, and then a completely fluorinated surface covered by -TiOF_2_ will be formed. Under the action of high concentration HF, These -TiOF_2_ will further react with HF to produce oxygen vacancies, as shown in Figures 1a–d (Wang et al., 2011). Surface fluorinated TiO_2_ prepared by post-treatment in liquid phase method often contains both surface adsorbing and inner surface phase doping fluorine. Researchers should comprehensively consider the fluorination effect and better understand the influence of inner surface phase doping fluorine in photocatalyst and distinguish it from the influence of surface adsorbing fluorine.

### 2.2 Lattice-doped fluorination

The radius of F^−^ (0.133 nm) is close to that of O^2-^ (0.132 nm), and F^−^ has a strong bonding ability with titanium atoms, so it is easier for F^−^ to stably dope TiO_2_ than other elements (Wardman, 1989). As mentioned in the previous section, when TiO_2_ is corroded by HF, lattice fluorine doping can be introduced while surface fluorine adsorption is achieved, but such lattice fluorine doping only exists in a few atomic layers on the surface and cannot enter the material phase. The realization of fluorine doping in the internal phase lattice of materials often requires the introduction of fluorine in the preparation process of TiO_2_ for *in-situ* synthesis. As the commonly used synthesis method, sol-gel method usually involves the nucleophilic reaction of fluorine ions in the hydrolysis process of titanium salts, and then is included in the material phase.

As shown in Figures 1e,f, according to the different number of titanium atoms coordinated with fluorine, Wang et al. proposed that fluorine exists in F-TiO_2_ in three forms: surface Ti_1_-F bond formed through replacing OH^−^ by F^−^; 2-bridged fluorine F2c (Ti_2_-F) and 3-coordinated fluorine F3c(Ti_3_-F) by substituting F atoms for O atoms (Wang et al., 2013). Due to possessing large number of lattice F atoms which could be converted into the lattice F3c atoms in the bulk TiO_2_ phase during the preparation processes, TiOF_2_ and HTiOF_3_ are reported to be the promising intermediates to synthesis anatase TiO_2_ (Liu et al., 2012). Hu et al. also reported the characterization of fluorine species such as Ti_1_-F, Ti_2_-F and Ti_3_-F in TiOF_2_/TiO_2_ composites by solid-state nuclear magnetism (Hu et al., 2020a).

## 3 Characterization of fluorine in TiO_2_

In general, X-ray photoelectron spectroscopy (XPS) and solid-state nuclear magnetic resonance (NMR) are used to characterize fluorine-modified TiO_2_ to determine the presence of fluorine species.

### 3.1 Analysis of fluorine species by X-ray photoelectron spectroscopy (XPS)

There are usually two F_1s_ peaks in the XPS spectrum of fluorinated TiO_2_ materials (Figure 1g), respectively in the range of 684.4–685.3eV (attributed to the physical adsorption of Ti_1_-F or the presence of TiOF_2_-like F (Ti_2_-F) in the material). And in the 687.8–688.6eV range (attributed to F^−^, which is substituted for O^2−^ into the lattice by either alone or co-doped with other elements (Yu et al., 2002). As shown in Figure 1h, Li et al. observed in the F_1s_ XPS spectra of typical FT powder and pure TiOF_2_ prepared by treating TiO_2_ with HF, that pure TiOF_2_ had a symmetric peak at 685.3eV, attributed to the Ti_2_-F atoms in TiOF_2_, and the peak at 687.8eV was attributed to the doped fluorine atoms in TiO_2_ (Li et al., 2005). Yang et al. also observed a symmetry peak at 684.5eV on F_1s_ XPS of anatase single crystal synthesized by TiF_4_ and HF, which could not be accurately attributed to either TiOF_2_ (Ti_2_-F) or surface adsorbed F (Ti_1_-F) (Yang et al., 2008). Wang et al. believe that the binding energy of F_1s_ is related to the coordination state of F-Ti, and the peak near 687.6eV on the XPS spectrum of F_1s_ can be attributed to the 3-coordination F (Ti_3_-F). However, since the fluorinated surface of Ti_2_-F is more stable, and the test depth of XPS is generally about 5–10 nm, the binding energy of F_1s_ can be classified into 3 Ti_3_-F. The surface of TiO_2_ fluoride synthesized by hydrothermal or sol-gel method is often unable to detect the peak near 687.6eV, but after Ti_3_-F is exposed to the sample surface by NaOH treatment, the signal of Ti_3_-F near 687.6eV can be detected by XPS. Therefore, considering the fuzzy allocation of F_1s_ signals in XPS and the detection limit of XPS in the bulk phase, additional characterization techniques are needed to clearly distinguish fluorine species (Wang et al., 2013).

### 3.2 Nuclear magnetic resonance (NMR) to study the Ti-F coordination

Because of its high natural abundance, high sensitivity and wide chemical shift range, ^19^F NMR is suitable for qualitative analysis of fluorine-containing compounds. Reyes-Garcia et al. studied the Ti-F coordination through solid-state ^19^F NMR testing, and they found TiO_5_F species in fluorine and boron co-doped TiO_2_ (Reyes-Garcia et al., 2007). After this, ^19^F NMR was used to study fluorine in F-doped TiO_2_(Hu et al., 2020a; Wang et al., 2022) and TiOF_2_/TiO_2_ mixtures (Hu et al., 2020a). Koketsu et al. tested solid ^19^F NMR to show that in sample Ti_0.78□0.22_O_1.12_F_0.4(OH)0.48_, fluoride ions near the vacancy were in three different chemical environments according to the coordination relationship between titanium atoms and vacancy (□): Ti_3_-F, Ti_2□1_-F and Ti_1□2_-F (Koketsu et al., 2017). The coordination environment of fluorine in the bulk phase can significantly affect the photocatalytic performance of TiO_2_. Wang et al. reported that Ti_3_-F with high 1s binding energy contribute to the enhancement of visible light activity of TiO_2_ fluoride. The introduction of such F leads to the formation of Ti^3+^, shrinks the band gap, and the presence of Ti_3_-F enhances the adsorption of hydroxyl. The photocatalytic activity was further improved (Wang et al., 2013). Subsequently, Hu et al. used NMR to study the Ti-F coordination of the sample TiOF_2_/TiO_2_ (Hu et al., 2020a). As shown in Figure 1i, multiple resonance signals at ∼ 15ppm can be attributed to the Ti_2_-F environment in the TiOF_2_ lattice, and the resonance at −84ppm can be attributed to the bulk phase Ti_3_-F. It was further confirmed that F was successfully incorporated into TiO_2_. After light treatment, the formation of a new signal at −151 ppm was attributed to the Ti_1_-F environment, indicating that the doped fluorine transformed from Ti_2_-F to Ti_1_-F and generated Ti^3+^ at the interface of TiOF_2_ and TiO_2_, which significantly enhanced the charge transfer efficiency in TiOF_2_/TiO_2_, thereby improving the photocatalytic performance. Therefore, according to the solid ^19^F NMR test results, fluorine atoms coordinate with different numbers of titanium atoms can be distinguished, but this research needs further exploration.

Furthermore, more comprehensive sample information can be provided by the combination of other technologies, such as electron paramagnetic resonance spectroscopy (Hu et al., 2020b) and electron energy loss spectroscopy (Wang et al., 2022).

## 4 Effect of fluorine on the photocatalytic hydrogen generation performance of TiO_2_

In the past years, fluorine-modified TiO_2_ has attracted attention in the field of photocatalytic hydrogen production (Wang et al., 2019; Bhom and Isa, 2024), which consists of the following steps: light absorption, charge separation and transport, and redox reactions at the photocatalyst’s surface.

### 4.1 Light absorption

**FIGURE 2:**
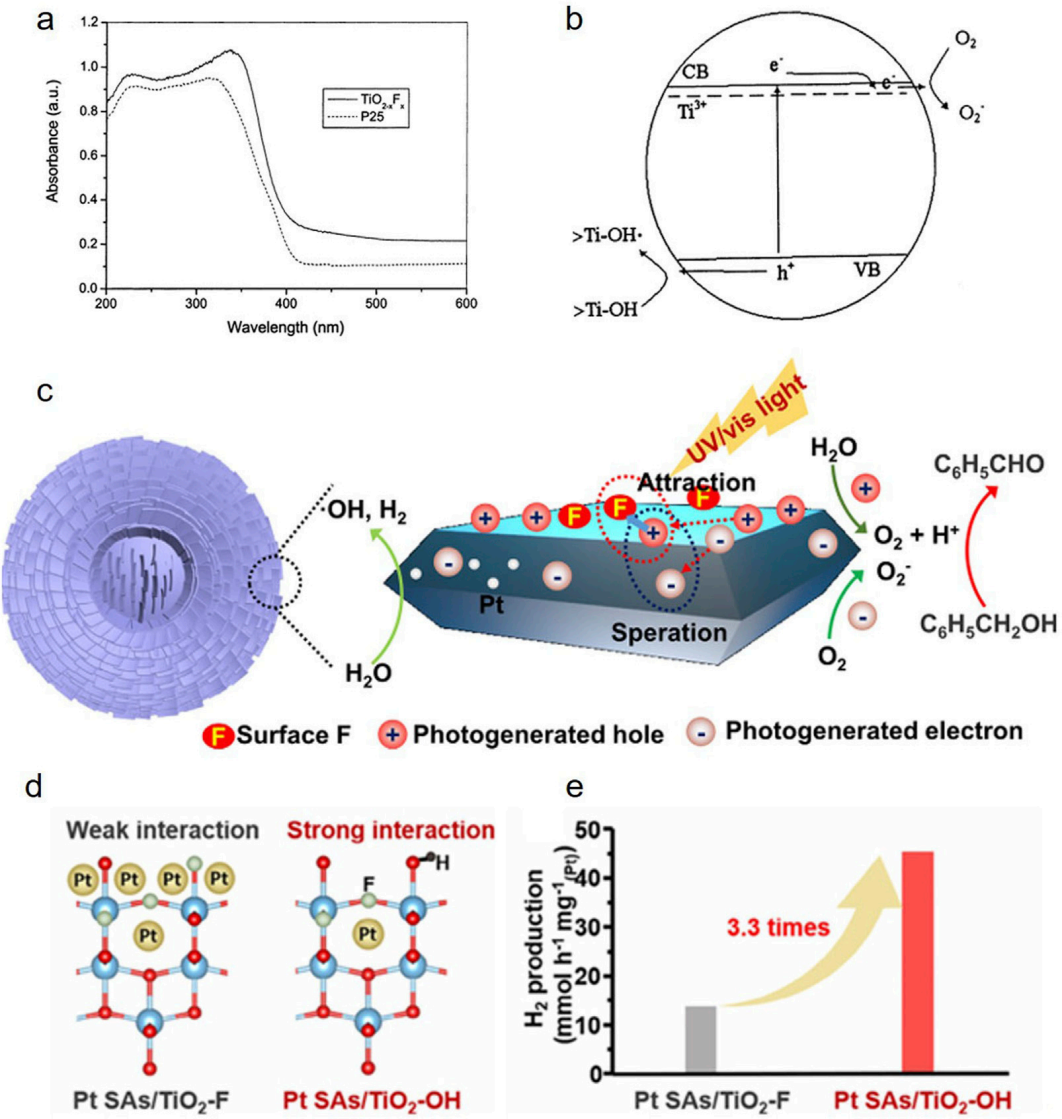
**(a)** UV-visible absorption spectra of Degussa P25 and the F-doped TiO_2_; **(b)** schematic energy level diagram for Ti^3+^ and charge-carrier dynamics in F-doped TiO_2_ (Yu et al., 2002); **(c)** proposed mechanism for the photocatalytic properties of TM-S (Hu et al., 2020b); **(d)** structure models and **(e)** normalized photocatalytic H_2_ evolution rate of Pt SAs/TiO_2_-F and Pt SAs/TiO_2_-OH (Wu and Schmuki, 2023).

Fluorinated TiO_2_ photocatalysts show stronger UV-visible light adsorption with a red shift (Figure 2a) were developed by Yu et al. through hydrolysis of titanium tetraisopropoxide in a mixed NH_4_F-H_2_O solution (Yu et al., 2002; Chen et al., 2022; Hou et al., 2024). The reduction of Ti^3+^ from Ti^4+^ by charge compensation of F doping form a donor level between the band gaps of TiO_2_ may benefit to the enhanced light absorption (Figure 2b). In addition, surface fluoridation also produces some oxygen vacancies, resulting in visible-induced photocatalytic activity. Le et al. used the thermal shock method to fluoridate TiO_2_ P25 powder at different temperatures, and the fluoridated sample produced oxygen vacancy at 400°C–600°C, which was confirmed by XPS spectroscopy as the formation of TiO_2_ surface fluoridation (Khoa Le et al., 2012).

Zhao et al. concluded that the surface lattice F3c atoms (Ti_3_-F) with higher 1s binding energy are identified to be the origin of visible light activity by analyzing the 1s CLSs of various types of F atoms in the fluorinated TiO_2_ (Wang et al., 2013). Further analyzing the electronic structures of the fluorinated TiO_2_ using semi-local density functional theory and non-local hybrid density functional theory calculations demonstrates that the introduction of the 3-coordinated surface F atoms leads to the formation of Ti^3+^ ions in the sub-surface, which is the cause for the bandgap shrinking, increasing the visible-light activity. However, the photocatalytic efficiency of fluorinated TiO_2_ for water splitting is limited due to the limited absorption under visible light irradiation and the high recombination rate of photogenerated electron-hole pairs (Yu et al., 2010; Li et al., 2020). Developing a method to synthesize F-TiO_2_ materials that with considerable visible-light photocatalytic activity is still a challenge.

### 4.2 Carriers separation and transport

Several investigations have been reported for increasing the efficiency of carriers separation/transport in TiO_2_ based materials through fluorine modified. Surface fluorination of TiO_2_ can significantly change the physicochemical properties and structure of the material surface: increasing the surface electronegativity, promoting the separation and transfer of surface charge, and inhibiting the recombination of electron hole pairs; promoting the formation of hydroxyl free radical and other active reactive substances (Yuan et al., 2025). The oxygen vacancy defects and Ti^3+^ centers formed on the surface of TiO_2_ during fluorination process also favor the separation of charge carriers (electrons and holes) and can trap the holes (Wang et al., 2021).

The surface charge separation can be further enhanced by loading Pt, Ag, Pd and other precious metals as cocatalyst on the fluorinated TiO_2_ (Vaiano et al., 2018; Díaz-Sánchez et al., 2021). Yu et al. reported that the F ions on the surface of TiO_2_ can greatly decrease the recombination rate of photogenerated carriers by acting as an electron-trapping sites to trap the photogenerated electrons due to its strong electronegativity and then transfer electrons to the Pt loaded (Yu et al., 2010). As shown in Figure 2c, our previous work further proved that the surface F anions with negative electric will attract the holes to migrate to the surface of TiO_2_ and inhibit the migration of photogenerated electrons, which further prevents electron-hole recombination (Hu et al., 2020b). Besides, the introduction of surface fluorine provides anchoring sites for Pt nanoparticles and strengthens the interaction between Pt nanoparticles and the TiO_2_ substrate resulting in significantly improved catalytic performance (Ji et al., 2019). Many recent works focus on the loading of metal single atoms (SAs) on TiO_2_ as cocatalyst for photocatalytic reactions (Hejazi et al., 2020; Cha et al., 2022). For example, Wu et al. reported that both surface and lattice Ti^3+^ suitable for Pt anchoring and charge compensation can be generated in pristine TiO_2_-F nanosheets with surface terminal F species. After surface F species are removed by NaOH treatment, Pt single atoms (SAs) were stabilized by lattice F (Figure 2d), and shows much higher photocatalytic hydrogen generation efficiency than Pt SAs on TiO_2_-F (Figure 2e (Wu et al., 2023; Wu and Schmuki, 2023). Recently, combined with the surface stabilizing effect of the as-formed F-C/F-Ti bonds, single-atom catalysts (Pd, Ir, Pt) on TiO_x_N_y_ nanorods surface via *in situ* fluoride ion etching for hydrogen evolution could be obtained (Zeng et al., 2025).

The crystallinity of fluorine-doped TiO_2_ could be improved upon F^−^ doping and then benefit to the higher bulk electronic conductivity, which is responsible for enhanced water splitting (Fang et al., 2014). Next, Hu et al. simulated the geometric structures and calculated the deformation density of the Ti_2_-F, Ti_3_-F, and Ti_1_-F sites, respectively. The neighboring Ti atoms of Ti_1_-F sites got more electrons, compared with those on theTi_2_-F or Ti_3_-F sites. The generation of terminal Ti_1_-F in TiOF_2_/TiO_2_ moved more electrons toward the terminal F atom resulting in the acceleration of the interfacial charge transfer (Hu et al., 2020b).

## 5 Conclusion

The current minireview focuses on the investigation of the surface-adsorbed fluorination and lattice-doped fluorination for F-TiO_2_ nanomaterials, and the role of fluorine in photocatalytic water splitting. According to the different number of titanium atoms coordinated with fluorine, the F atoms introduced to fluorinated TiO_2_ system are classified into terminal Ti_1_-F, bridging Ti_2_-F and Ti_3_-F. In conclusion, both surface-adsorbed fluorination and lattice-doped fluorination are effective measures to improve the photocatalytic performance. Fluorine ions on the surface of TiO_2_ can significantly change the physicochemical properties and structure of the material surface: increasing the surface electronegativity, promoting the separation and transfer of surface charge. Especially, the surface Ti_3_-F is identified to be the origin of visible light activity. The surface lattice Ti_2_-F are beneficial to stabilize Pt SAs and then bring high photocatalytic efficiency. Defects such as surface Ti^3+^ and oxygen vacancy defects formed during fluorination process could change the local electronic structure and improve the photocatalytic performance. Ti^3+^ defects introduced by lattice-doped fluorination can regulate the band structure of TiO_2_ and inhibit photogenerated carrier recombination. The generation of terminal Ti_1_–F moved more electrons toward the terminal F atom resulting in the acceleration of the interfacial charge transfer.

Although great progress has been made in the role of fluorine in photocatalysis, there are still many problems that need to be fully studied further. For example, due to the varied fluorine species in fluorine-modified TiO_2_, there are challenges in the precise regulation of doped fluorine species, and the mechanism of action of various doped fluorine species on the improvement of photocatalytic performance at the atomic scale also needs to be improved.
